# Continuous glucose control in the ICU: report of a 2013 round table meeting

**DOI:** 10.1186/cc13921

**Published:** 2014-06-13

**Authors:** Jan Wernerman, Thomas Desaive, Simon Finfer, Luc Foubert, Anthony Furnary, Ulrike Holzinger, Roman Hovorka, Jeffrey Joseph, Mikhail Kosiborod, James Krinsley, Dieter Mesotten, Stanley Nasraway, Olav Rooyackers, Marcus J Schultz, Tom Van Herpe, Robert A Vigersky, Jean-Charles Preiser

**Affiliations:** 1 Department of Anesthesiology and Intensive Care Medicine, K32, Karolinska University Hospital, Stockholm, Huddinge 14186, Sweden; 2 GIGA - Cardiovascular Sciences, University of Liege, Institute of Physics, B5, Allee du 6 aout, 17, Liege 4000, Belgium; 3 The George Institute for Global Health and Royal North Shore Hospital, University of Sydney, St Leonards, Sydney, NSW 2065, Australia; 4 Department of Anesthesia and Intensive Care Medicine, OLV Clinic, Aalst 9300, Belgium; 5 Starr-Wood Cardiac Group, 9155 SW Barnes Road, Portland, OR 97225-6629, USA; 6 Department of Medicine III - Division of Gastroenterology and Hepatology, Medical University of Vienna, Waehringer Guertel 18-20, Vienna 1090, Austria; 7 University of Cambridge Metabolic Research Laboratories, Level 4, Wellcome trust MRC Institute of Metabolic Science, Box 289, Addenbrooke’s Hospital, Hills Road, Cambridge CB2 0QQ, UK; 8 Jefferson Artificial Pancreas Center and Anesthesiology Program for Translational Research, Department of Anesthesiology, Jefferson Medical College of Thomas Jefferson University, 1020 Walnut Street, Philadelphia, PA 19107, USA; 9 Saint-Luke’s Mid America Heart Institute, University of Missouri - Kansas City, 4401 Wornall Road, Kansas City, MO 64111, USA; 10 Division of Critical Care, Stamford Hospital and Columbia University College of Physicians and Surgeons, 30 Shelburne Road, Stamford, CT 06904, USA; 11 Department of Intensive Care Medicine, University Hospitals Leuven, Herestraat 49, Leuven B-3000, Belgium; 12 Surgical Intensive Care Units, Tufts Medical Center, 800 Washington Street, NEMC 4360, Boston, MA 02111, USA; 13 Anesthesiology and Intensive Care Clinic, Karolinska Institute and University Hospital, Huddinge 14186, Sweden; 14 Department of Intensive Care Medicine, Academic Medical Center at the University of Amsterdam, Meibergdreef 9, Amsterdam 1105 AZ, The Netherlands; 15 Department of Intensive Care Medicine, University Hospitals Leuven, Herestraat 49, Leuven B-3000, Belgium; 16 Department of Electrical Engineering (STADIUS) - iMinds Future Health Department, Katholieke Universiteit Leuven, Leuven, Heverlee B-3001, Belgium; 17 Diabetes Institute, Walter Reed National Military Medical Center, Bethesda, MD 20895, USA; 18 Department of Intensive Care, Erasme Hospital, Université libre de Bruxelles, 808 route de Lennik, Brussels 1070, Belgium

## Abstract

Achieving adequate glucose control in critically ill patients is a complex but important part of optimal patient management. Until relatively recently, intermittent measurements of blood glucose have been the only means of monitoring blood glucose levels. With growing interest in the possible beneficial effects of continuous over intermittent monitoring and the development of several continuous glucose monitoring (CGM) systems, a round table conference was convened to discuss and, where possible, reach consensus on the various aspects related to glucose monitoring and management using these systems. In this report, we discuss the advantages and limitations of the different types of devices available, the potential advantages of continuous over intermittent testing, the relative importance of trend and point accuracy, the standards necessary for reporting results in clinical trials and for recognition by official bodies, and the changes that may be needed in current glucose management protocols as a result of a move towards increased use of CGM. We close with a list of the research priorities in this field, which will be necessary if CGM is to become a routine part of daily practice in the management of critically ill patients.

## Review

## Introduction

Achieving adequate glucose control in ICU patients is complex and difficult to perform optimally. Until relatively recently, intermittent blood-gas analyzer and central laboratory measurements of blood glucose from arterial blood samples have been the only means of monitoring blood glucose levels [1]. However, intermittent measurements are limited by the workload associated with the sampling process and the potential that between-measurement events may be missed. With growing interest in the possible beneficial effects of continuous over intermittent monitoring and the development of several continuous glucose monitoring (CGM) systems, a round table conference was convened in March 2013 to discuss and, where possible, reach consensus on various aspects related to glucose monitoring and management. Leading experts in the field of glucose control in ICU patients and invited members of interested industry companies joined for presentation and discussion. After the meeting, a draft report was circulated to all participants by email for critical review. Representatives of the invited industry companies were asked to include a brief summary of their devices in the additional file of this report (Additional file 1), but, other than participation in the open discussion periods of the meeting, had no influence on content.

## Continuous glucose monitoring

### Definitions

CGM has been proposed as a means to improve management of dysglycemia. Although termed ‘continuous’, current systems still sample intermittently, with a measurement interval of a few milliseconds up to 15 minutes. Some systems average the frequent intermittent measurements and display them as a single reading or moving average, updated regularly. Nevertheless, such measurements can be considered as having ‘real-time’ value especially when compared to their intermittent counterparts, although physiological or data processing lag time may be present depending on the sampled body fluid. Two factors can be considered when defining ‘continuous’: the frequency of actual glucose measurements and the immediacy of the data display. Clearly, measurements need to be frequent enough to capture all glucose dynamics. Based on current knowledge of the physiology of glucose and insulin metabolism in non-critically ill patients [2], an interval of 10 to 15 minutes between measurements is the likely maximum interval that would detect most glycemic dynamics, although faster dynamics may be observed when parenteral nutrition is modified and particularly when an intravenous glucose bolus is administered. The Clinical and Laboratory Standards Institute (CLSI) guidelines use 15 minutes as the cutoff for their definition of continuous monitoring [3], but which cutoff should be used to separate ‘continuous’ from ‘frequent intermittent’ sampling is debatable. More data on glucose trends in the critically ill are needed before clinically relevant sampling frequencies can be defined. The real-time output of CGM devices should be as instantaneous as possible, although there will generally be a lag period, the duration of which will depend on the site and frequency of sampling and data processing. The continuous display enables trends to be identified and visualized.

Importantly, the purposes of any such device are to improve clinically relevant outcomes and to reduce associated nursing workload and ideally costs. Although the overall accuracy of many CGM systems is less than that of intermittent systems using central laboratory testing [4], this limitation is to some degree mitigated by the ability to follow the direction of change in glucose levels, theoretically allowing earlier intervention to maintain blood glucose concentrations within acceptable ranges. A less-often cited advantage is the decreased need for multiple finger-pricks or blood pulls with a continuous system, which may reduce patient discomfort and nurse workload [5,6].

Several CGM systems are now available for clinical use and early results from clinical trials in critically ill adults [7-14] and children [15,16] have been published. However, no studies have assessed clinical outcomes using the continuous approach compared to an intermittent system; furthermore, the different sensors used, the different comparators, and the lack of standardized performance metrics make it difficult to compare results.

### Overview of techniques for glucose measurement

The three predominant techniques currently used for continuously measuring glucose levels in the ICU involve glucose oxidase, mid-infrared spectroscopy and fluorescence.

The glucose oxidase technique is based on the sensing of hydrogen peroxide (H_2_O_2_) released when glucose is converted to glucolactone: the greater the concentration of glucose, the more H_2_O_2_ will be released and the stronger the signal. Results can be influenced by interference from molecules other than glucose (for example, uric acid, acetaminophen and salicylic acid) which oxidize the H_2_O_2_.

Mid-infrared spectroscopy detects an absorption spectrum for glucose in plasma using different wavelength filters.

Fluorescence techniques rely on quenched chemical fluorescence to measure glucose concentration [17]. Fluorescence glucose sensors are associated with a foreign body response, are sensitive to local pH and/or oxygen, and require a light source.

### Monitoring sites: clinical experience

Glucose can be measured in whole blood, plasma, interstitial fluid, and microdialysis fluid and values will vary according to which fluid is used [18]. Generally, plasma glucose is considered the ‘gold standard’. Glucose dissolves in water and because plasma has a higher water concentration (approximately 93%) than do red blood cells (approximately 71%), plasma will have a higher glucose concentration than will whole blood. The difference in laboratory-measured glucose concentration between whole blood and plasma will also vary with the hematocrit. Because some glucose diffuses from the plasma to interstitial fluid and tissues as blood circulates through the capillary system, arterial blood glucose is usually higher than venous glucose. Arterial blood glucose and capillary blood glucose are generally similar, although when blood glucose levels change rapidly, there may be a delay before similar changes are seen in capillary blood. Microdialysis concentrations tend to be slightly lower than those present in the surrounding tissue or blood.

The degree of invasiveness of a CGM technique varies from highly invasive (for example, intravascular devices) through the minimally invasive subcutaneous techniques, to non-invasive transdermal devices. Although studies comparing the accuracy and performance of more versus less invasive CGM systems have not yet been performed, preliminary data suggest that, moving through the spectrum from invasive to non-invasive, accuracy generally decreases as does the risk of complications, including infections. The type of monitor selected should be adjusted to patient characteristics, including the severity of illness of the patient and the type of access available. For example, a severely ill, unstable ICU patient will likely already have arterial and/or central venous lines *in situ* allowing invasive intravascular monitoring, whereas a stable patient ready for ward transfer can be monitored using a less- or non-invasive device. Moreover, severely ill patients are more likely to be receiving mechanical ventilation and/or sedative agents, making clinical symptoms of hypoglycemia more difficult to detect and perhaps arguing in favor of the more accurate invasive devices. When comparing devices it is essential to state which reference measurement technique is used so that results can be easily compared. Whenever possible, arterial glucose measurements with a blood gas analyzer or by a central laboratory should be used as the comparator as these are the most accurate and reproducible [1]; when this is not possible, or when the device under study uses venous sampling, venous blood glucose should be used as the comparator. When venous sampling is used, the specific vessel should be defined.

Intravascular CGM devices can be divided into three groups: (1) those that have an intravascular sensor inserted into the lumen of an artery or peripheral/central vein and directly measure the blood glucose concentration without consuming blood in the process; (2) those in which a small blood sample is taken from the intravascular catheter and passed over an external sensor; and (3) those in which a blood sample is re-circulated after passing through an external sensor without blood loss. The accuracy of intravascular microdialysis probes will vary according to their position - for example, if integrated into the central venous catheter, a much larger membrane will be possible than if positioned in a smaller peripheral vein catheter, allowing a greater area for equilibration and a more rapid and reliable result [19]. Recent studies using a central venous catheter with a microdialysis membrane have demonstrated good agreement between microdialysis glucose measurements and reference venous and arterial blood gas values in patients undergoing major abdominal surgery or cardiac surgery [20,21].

Interstitial fluid glucose is generally measured with subcutaneous probes, often inserted on the abdominal wall or upper arm. Interstitial fluid glucose levels depend on the rate of glucose diffusion from plasma to the interstitial fluid and the rate of uptake by subcutaneous tissue cells; hence, they are influenced by blood flow, the metabolic rate of adjacent cells, capillary permeability, degree of hydration or edema, and so on, all of which may be altered in critically ill patients [18]. Several subcutaneous devices have been tested in critically ill patients and have been shown to have good agreement with reference arterial and venous samples [12,22-24]. Moreover, similar accuracy has been reported in critically ill patients with and without shock requiring norepinephrine therapy [22], and in cardiac surgery versus non-critically ill patients [25,26]. Nevertheless, the accuracy of interstitial fluid monitoring needs to be further investigated, in particular in unstable patients. One concern with subcutaneous interstitial fluid probes is the tissue trauma created during insertion, such that measurements may be less accurate for several hours after insertion. There is a time lag between change in blood glucose and that measured in the interstitial fluid, which is, however, unlikely to result in ineffective treatment in case of an emerging hypo- or hyperglycemic event [27-30]. The clinical relevance of this time-lag needs to be contrasted against current practice with a typical delay of 5 to 10 minutes to take the sample and to measure glucose on an analyzer.

Transcutaneous devices are also being developed. One such device uses a biosensor that can measure transdermal glucose flux, which is proportional to the blood glucose concentration. The skin is prepared by microabrasion to remove the dead surface cells and the biosensor then applied, using the glucose oxidase reaction to create a measureable signal for interstitial glucose. In pilot studies of cardiac surgery patients, good agreement with peripheral blood was demonstrated [31,32].

All techniques have limitations related in part to the sampling site used (venous, arterial or capillary blood, plasma, and interstitial fluid) [18], but also to the need for anticoagulation with some intravascular devices, problems of local inflammation, and need for recalibration. Rice and Coursin [33] recently proposed a list of attributes for the ‘ideal’ CGM system (Box 1). For all CGM systems, specific performance characteristics related to the clinical utility of the system need to be clearly defined (Box 2).

### Trend accuracy versus point accuracy

One of the key advantages of CGM systems is their ability to identify and display trends in blood glucose measurement. Hence, when considering the performance of these devices, additional metrics may need to be developed to complement current assessment of accuracy. Point accuracy is defined as the difference between the current displayed blood glucose value and the current true blood glucose value. Trend accuracy is defined as the degree to which an estimate of the rate of change in blood glucose concentration over a given time interval approximates the true rate of change. Further research is required to establish the duration over which trend accuracy should be calculated and the relative importance of point accuracy versus trend accuracy in terms of clinical outcomes.

**Figure 1:**
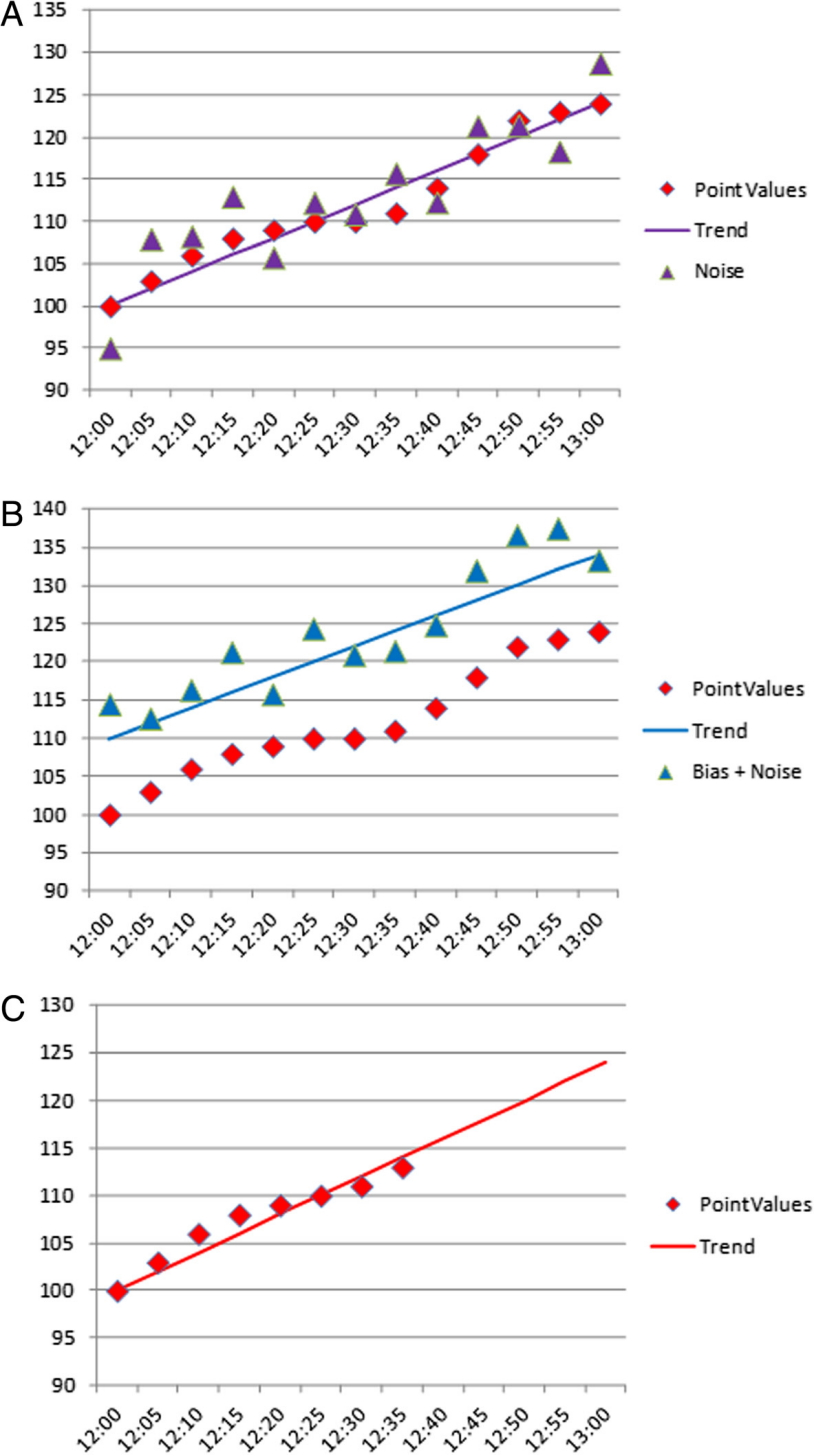
**Schematic representation of the potential advantages of using trends. (A)** If imprecision or noise is random or normally distributed, the trend line will filter it out. **(B)** If the measurement system has a fixed bias, trend will not be affected but individual values could be. **(C)** When trying to predict future events, trend may be clinically more important than the current absolute blood glucose value.

In theory, the use of trending could have several potential advantages over individual values (Figure 1), including: that it is less sensitive to random noise, because, if present, noise will be filtered out by the trend line, at least when the period used to calculate the trend is long enough; there is little effect of bias - the presence of a constant over- or under-shoot of the value will not affect the trend. However, there are also several disadvantages. First, there is a lag time when calculating the trend that will depend on the frequency of sampling and the number of measurements and time-lapse over which those measures are used. With longer time intervals between measurements, trending will reflect real changes less accurately, certainly when changes are rapid and intervals are long. Second, if there is a lag time or a bias, extrapolation of the trend line can amplify the error. Third, most current glycemic control protocols rely on PID (proportional, integral, derivative) control with insulin rates determined as a function of the current blood glucose (P), accumulation of historical blood glucose values (I), and the trend (rate of change) in blood glucose (D). Hence, for current protocols, all three aspects need to be accurate; it is not sufficient just to have accurate trend accuracy - point accuracy also needs to be good.

Thus, at the present time, both good point accuracy and good trend accuracy are required to achieve optimal glycemic control. However, the more continuous the measurement, the clearer and more reliable the trend will become. In the future, use of algorithms designed specifically for CGM may also reduce the need for highly accurate point measurements. The period of time over which trend should be assessed will depend on lag time and may also depend on the type of patient.

### Standards for reporting performance

Standards for reporting of clinical trials of CGM systems need to be developed so that results can be easily compared. In this context, we can consider factors related to the patients and the device and those related to the impact of the device on clinical outcomes. In terms of the device and patient, several aspects need to be reported regarding demographics (age, gender, comorbidities, including diabetes, disease severity), use of vasoactive drugs, design (single-center versus multicenter, type of center, number of samples, comparator), glucose targets (target range), definition of hypoglycemia and hyperglycemia, time in range, analytical and clinical accuracy, number of patients unable to monitor and reasons, down-time (time needed for calibration when no signal/reading available) and time to display, and safety (bleeding events, infections, outliers, alarm performance).

In terms of characterization of accuracy of the system being tested versus the comparator, the Bland-Altman plot remains indispensable, showing the difference between the two measurements either as a function of the average of the two measurements or, when there is a ‘gold standard’, as a function of the comparator [34]. The 95% confidence interval (1.96 × standard deviation) of a tested blood glucose meter against a gold standard can be deduced from these plots. Various grid systems have also been proposed, of which the Clarke error grid [35] is currently the most widely used. However, this grid was not designed for CGM systems and does not reflect rapid changes in the blood glucose level or account for potential errors in insulin dosing. As such, the so-called continuous glucose error grid analysis (cEGA) has been proposed, which is designed to capture errors in the rate and direction of change in glucose between measurement methods [36]. This technique, initially developed for outpatient care, is an interesting approach but relatively complex, requiring specific software and frequent sampling [37]. The R-deviation is another potential metric to assess the accuracy of CGM systems [38]. This value is a numerical metric of rate of change accuracy, based on the deviation between the rates of change in reference and test sensor glucose fluctuations.

How to report on the impact of a device when used with a treatment protocol is perhaps less clear. For this purpose, three domains of glycemic control can be considered: hyperglycemia, hypoglycemia, and glycemic variability [39]; glucose complexity has been suggested as a possible fourth domain [40]. The three domains are all associated with increased mortality in critically ill patients [39] and, as such, the number and duration of hypo- and hyperglycemic episodes (using pre-specified parameters), the time in target, the degree of glucose variability (and possibly complexity) should all be reported when assessing the clinical impact of a new device, in addition to clinical outcomes, including mortality and morbidity measures. Further study is needed to determine how best to define trend and hypoglycemia (including sensitivity and specificity) for regulatory approval (see below).

### Alarms, warning signals

**Figure 2:**
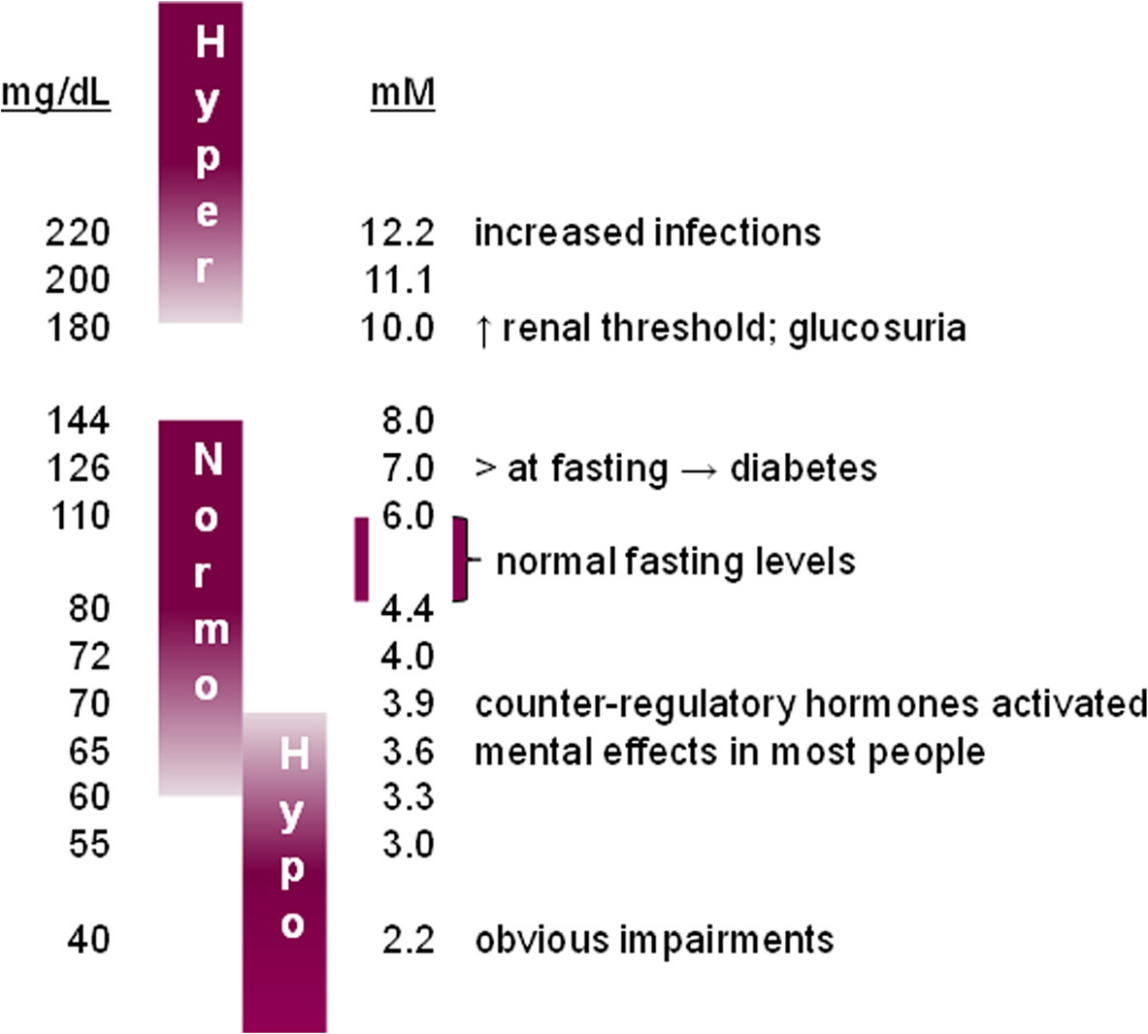
The clinical impact of hypo/hyperglycemia varies according to the degree away from normal values.

Alarms on CGM systems generally concern the three domains of glycemic control listed earlier. Determining at which value alarms should be set for each domain remains difficult. The clinical impact of hypo/hyperglycemia will vary according to the degree and time away from normal values (Figure 2), with considerable overlap among individuals. Several studies have suggested that patients with acute coronary syndromes and severe brain injury may be more sensitive to low blood glucose levels [41,42], at least in the absence of tight glucose control [43]. Therefore, in some groups of critically ill patients, target glucose ranges may need to be set higher than in other groups. Generally, a blood glucose ≤40 mg/dl is considered to represent severe hypoglycemia [1,44] and a level of 41 to 70 mg/dl moderate hypoglycemia [1], but studies have used different definitions. Hyperglycemia is variably considered as values >140 or 180 mg/dl. Glycemic variability is even more difficult to define; a relatively high value of the coefficient of variation of >20% has been suggested to define high variability, because it is associated with worse outcomes than values <20%. Variability is also related to ongoing therapy. Glycemic targets will also vary according to individual patient characteristics, including age, comorbidities (notably diabetes), type of patient (for example, surgical versus medical), and so on. Alarm settings therefore need to be adjustable for individual patients. Further study is needed to define optimal target ranges for different groups of patients and to clarify the impact of alarms on clinical practice and patient outcomes. With the development of better validated CGM systems and better knowledge of glucose trends in the critically ill, alarms for trend changes will be developed and have the potential to prevent hyper- and hypoglycemia. Predictive alerts are already in use on some devices inserted subcutaneously.

### Criteria for approval by the official bodies

In terms of safety and effectiveness, it is unclear which metrics should be used to indicate sufficient accuracy and reliability. The CLSI has produced new standards for point-of-care testing [45]; however, these standards may not be applicable to CGM systems. In our 2013 Consensus document, we suggested that the minimum standard for glucose meters to be used in critically ill patients should be that 98% of readings should be within 12.5% of a reference standard (or within ±10 mg/dl for readings <100 mg/dl); the remaining 2% of readings should be within 20% of a reference standard [1]. The mean absolute relative difference should be cited and values should be <14%; values >18% are considered to represent poor accuracy. For trend accuracy there is not yet an accepted metric. The R-deviation may be useful, but further study is needed [38]. Other concerns that need to be addressed include signal stability, drift, variability, and drop-out; potential interferences (for example, acidosis, hematocrit, bilirubin, hemoglobin, medications and intravenous fluids); edema and nutritional status; and number and characterization of outliers. As yet, there are no clearly defined metrics for reporting what is sufficient in terms of accuracy and reliability.

## Insulin algorithms

An algorithm can be defined as ’a formalized sequence of instructions for solving a complex problem in finite processing steps’ [46]. Algorithms in the field of tight glucose control are used to standardize care, for quality assurance and to avoid intuitive decision making. An optimal system should be accurate, safe, efficacious, simple to use, reliable, flexible for different patient populations, assessable in real-time, fit into workflow, require a low number of glucose measurements (if not based on CGM), and take into account inter- and intrapatient variability and carbohydrate intake. Algorithms should incorporate dynamic scale protocols, instead of static sliding-scale protocols [47]. Although early algorithms were paper-based [48,49], increasingly, glucose control algorithms are computer-based, enabling more complex protocols to be developed. Several studies have demonstrated improved glucose control with computer-based compared to paper-based algorithms [50-52]. A common type of algorithm is the PID algorithm in which deviation of the blood glucose value from the target range is corrected by adjusting the dose of insulin using a linear combination of absolute deviation, trend, and the sum of past deviations [53]. Another main type of glucose algorithm used in critical care is the model-based or model-predictive control algorithm, which adjusts insulin dose according to a mathematical model of the relationship between blood glucose and insulin [54,55]. Better standardization of algorithm development is needed [56].

Many algorithms for glucose control have been developed. Wilson and colleagues [57] identified 12 different algorithms and, using blood glucose records from an actual hyperglycemic patient, calculated the insulin doses that would have been recommended by each protocol. There was considerable variability among protocols in patterns and ranges of recommended insulin dose (range 27 to 115 units), and adjustments to dose when nearing target glucose. Protocols therefore behave differently and may have greater influence on outcomes than the glucose measurement error. Different algorithms may be better suited to various patient populations or clinical settings.

Clinical testing and comparison of algorithms is resource intensive in terms of patients, staff, time, and costs. Moreover, the majority of algorithms for glycemic control in the ICU are based on intermittent glucose measurements and new algorithms will need to be designed if CGM systems become more widely used. When comparing algorithms, standard glucose-centric outcomes need to be reported, including numbers of hypo- and hyperglycemic episodes. One useful parameter that has been suggested is the cumulative time-in-band, which calculates the percentage of blood glucose values that fall within a specified range. This measure is independent of sampling frequency, can be applied to all algorithms and is simple to calculate; however, it is only useful when comparing algorithms that target the same blood glucose band.

*In silico* simulation models using ‘virtual’ ICU patients have been suggested to reduce some of the burden of clinical algorithm comparisons and to accelerate the assessment process. These systems can be used to optimize design parameters and safety features, test effects of changes in nutrition or other medications and interventions, and assess effects of measurement errors or delays. At least four currently available ICU simulators are known: the Cambridge [58], Virginia [59], Leuven [60], and Christchurch [61] models and simulation models are beginning to be used in the critical care setting of glycemic control. Wilinska and colleagues used simulation to compare the effects of different algorithms [62] and the performance of a newly proposed algorithm [63]; the study results were reproduced in the simulated populations. Although these systems need further study, it seems likely that the virtual patient will play an increasingly large role in the ongoing development of CGM systems and glycemic control protocols in the ICU setting.

The development of closed loop systems, which deliver insulin in a glucose responsive fashion every 1 to 15 minutes based on CGM measurements, is the most promising approach to improve glucose control once CGM becomes routinely available. Closed loop systems are being aggressively pursued and may help modulate glucose delivery to further reduce the risk of hypoglycemia. Automated closed-loop glucose control based on continuous subcutaneous glucose measurements and model predictive control in critically ill adults was associated with better glycemic control compared to a local sliding scale protocol [64].

## Priorities for research

The expert group defined eight areas where research should help to advance glucose monitoring in the near future to the likely benefit of critically ill patients. First, the different devices for CGM need to be better validated in terms of accuracy and reliability. Head-to-head comparisons are needed, particularly for devices sampling different compartments. Second, the clinical relevance of inaccuracies in glucose measurements should be shown in error grids adapted to current therapeutic algorithms. Third, glucose trends in critically ill patients and subgroups need to be more clearly characterized, so that better definitions of the rate of change can be developed and, thereby, the frequency of sampling needed to describe clinically relevant trends. Fourth, the effect of different insulin treatment algorithms on glucose variability should be studied with development of new and enhancement of existing glucose control protocols based on CGM. Fifth, development and validation of metrics for trend accuracy are required. Sixth, universal metrics to assess glycemic control and blood glucose variability that could be used with continuous as well as intermittent data should be defined and agreed upon. Seventh, at a later time-point, randomized controlled trials need to be conducted assessing the effects of CGM versus intermittent systems on outcome in critically ill patients, including assessment of patient-centered outcome measures (glycemic control and morbidity incidence). Eighth, closed loop systems for glucose control in critically ill patients should be developed and eventually validated and assessed in randomized controlled trials as above.

## Conclusion

CGM mandates the development of new approaches to the analysis of parameters of glucose regulation, such as glucose variability and glucose complexity, and also provides a tool to help effect these analyses. While CGM systems clearly have the potential to improve blood glucose control and patient outcomes, this remains a potential that has not yet been demonstrated in clinical practice. Future studies may be able to demonstrate real clinical benefits and reveal the optimal use of the different CGM systems (which system for which patient). When discussing how best to assess CGM, different goals can be considered, including maintenance of specified target levels, which may vary in different patient populations; avoidance of hypoglycemic events; assessment of glucose variability; and degree of glucose complexity. Most important, however, will be the impact of each device on clinical outcomes, including better glucose control and fewer hypoglycemic episodes; this is of far more relevance to clinicians and patients than small differences in accuracy.

## Box 1. Suggested criteria for the ideal continuous glucose monitoring system [33]

Rapid: very little lag between blood glucose and the measured value.

Accurate: each measurement should be within accuracy guidelines suggested recently [1].

Free of interference: minimal, if any, important interference, such as drugs or physiologic perturbations.

Inert: the sensor should not react with the tissue or form a coating rendering the device inaccurate over time.

Robust: the system must be able to perform within the dynamic and busy ICU and operative setting.

Minimally invasive

Cost-effective

## Box 2. Performance characteristics related to the clinical utility of continuous glucose monitoring systems that need to be clearly defined for each system

Frequency of sampling

Delay to display

Lag time

Biofilm development

Measurement accuracy

Reliability (time to sensor failure, frequency and duration of data gaps)

Need for and frequency of calibration

Ability to recognize and correct for interference

Automation

Need for anti-coagulation

Safety

Site of access

Handling of outlier values

Alarms

Clinical effectiveness (that is, impact on glucose control and prevention of hypoglycemia)

Cost-effectiveness

Possibility of combining glucose monitoring with other measurements

## Abbreviations

CGM: Continuous glucose monitoring; CLSI: Clinical and Laboratory Standards Institute; PID: Proportional, integral, derivative.

## Competing interests

JW has received grant support from CMA Microdialysis (now part of Maquet) payed to his university, Karolinska Institutet. TD has no conflicts of interest related to this manuscript. SF has received research funding and travel expenses from GluMetrics Inc., provision of equipment for research from Dipylon Medical (Eirus), travel expenses and consulting fees to his employer from Edwards, and provision of equipment for research to his employer from Nova Biomedical (StatStrip). LF has received funding for research, speaker’s fees and consultancy fees from Edwards Lifesciences. AF has received grant support from Sanofi, Edwards Lifesciences and Johnson & Johnson, has received speaker fees from Sanofi, Edwards Lifesciences, Johnson & Johnson, and Echo Theraputics, owns equity shares of Edwards Lifesciences, Medtronic, Sanofi, Glumetrics and Echo Therapeutics stock, and has served as a consultant for Edwards Lifesciences, Medtronic, Glumetrics, Glysure and Echo Theraputics. UH has received consultant fees from Medtronic. RH has received speaker honoraria from Medtronic, Lifescan, Eli Lilly, BBraun, and Novo Nordisk, served on advisory panels for Animas, Edwards Lifesciences, and Minimed Medtronic, received grant or material support from Abbott Diabetes Care, Animas, Edwards Lifesciences, Medtronic, received license fees from BBraun, served as a consultant to BBraun and Profil, and has patent applications. JJ has received company-funded research from Echo Therapeutics, Edwards Lifesciences LLC, DexCom Inc, Medtronic Diabetes, and GluMetrics Inc paid to his institution, Thomas Jefferson University. He has served on advisory boards for Echo Therapeutics Inc., Edwards Lifesciences LLC, and Medtronic Diabetes. MK has received research grants from Medtronic Minimed, Glumetrics, Maquet, Gilead Sciences, Genentech, Sanofi-Aventis, and the American Heart Association, and acted as a consultant or member of an Advisory Board for Medtronic Minimed, Gilead Sciences, Genentech, AstraZeneca, Abbvie, and Hoffman La Roche. JK has received Consulting/Advisory Board fees from Edwards Life Sciences, Medtronic, Glysure, and Optiscan Biomedical. DM has received reimbursements from Medtronic and BBraun paid to the University (KULeuven). SN has received speaker and consultant fees from Alere, Inc., consultant fees from Edwards LifeSciences, and stock options from Echo Therapeutics and OptiScan. OR has received grant support from CMA Microdialysis (now part of Maquet). MJS has received consultant fees from Medtronic Inc., GlySure Ltd, Edwards Life Sciences and Roche Diagnostics and financial support from Medtronic Inc. and OptiScan Biomedical - all fees and financial supports were paid to his institution. TVH has one patent in the related field. RAV has received investigator-initiating grants from DexCom and has served as a consultant for Medtronic Diabetes Care, Sanofi-Aventis, Bayer, Abbott Diabetes Care, and Roche. J-CP has received speaker fees from Edwards, Fresenius, Maquet and Optiscan. He has also served as a consultant for Edwards, Medtronic and Optiscan.

## Supplementary Material

Additional file 1 Summary of the current continuous glucose monitoring devices (provided by the industrial sponsors of the meeting, listed in alphabetical order). Click here for file
