# Supplementary material for: Continuous glucose control in the ICU: report of a 2013 round table meeting
Source: Crit Care. 2014 Jun 13;18(3):226. doi: 10.1186/cc13921 (PMC4078395; doi:10.1186/cc13921)
Supplement: Additional file 1 — Summary of the current continuous glucose monitoring devices (provided by the industrial sponsors of the meeting, listed in alphabetical order). [file cc13921-S1.docx]

**Additional file:** Summary of the current continuous glucose monitoring devices (provided by the industrial sponsors of the meeting, listed in alphabetical order).

**B Braun:** B Braun SpaceGlucoseControl (SGC) is a decision support system for insulin therapy fully integrated in the Space infusion pump system. It is indicated for critically ill patients in a closely monitored environment, for example, ICUs or operating rooms (ORs). The system recommends an insulin dose rate and measurement interval using a model predictive control algorithm based on the patient's actual and previous blood glucose levels and under consideration of the current carbohydrate feeding, which is automatically updated from enteral and parenteral nutrition pumps. Using SGC, glucose control can be significantly improved compared to manual control and effective glucose control can be established.

**EchoTherapeutics:** The Symphony® continuous glucose monitoring (CGM) system from Echo Therapeutics is a non-invasive, needle-free CGM system targeted for use with ICU patients. Symphony’s color display of graphical trend information updated every minute, and customizable low and high glucose alerts, combine to provide ICU staff with an early warning system for potential glucose excursions. Symphony’s transdermal approach to access interstitial glucose levels and its wireless transmission of glucose data from sensor to monitor minimize the risk of infection, inflammation, pain or discomfort that can be associated with other methods of glucose monitoring.

**Edwards:** The GlucoClear^TM^ CGM system from Edwards Lifesciences measures blood sugar by glucose oxidase sensing technology through in-blood measurement. Blood is automatically drawn and analyzed every 5 minutes, with real time graphical display. Blood is then returned to the patient and the system automatically self-calibrates. GlucoClear CGM has been designed for ICU and OR patients. The GlucoClear CGM is designed to be highly accurate. In a recent ICU study, it was shown to be at least 95%, 10/15% accurate with a mean absolute relative difference (MARD) of 5.05%. (10/15% is the proportion of the GlucoClear CGM’s values within ±10 mg/dL of Yellow Springs Instruments (YSI) (for YSI ≤66.7 mg/dL) and within ±15% of YSI (for YSI >66.7 mg/dL).) A 50-patient ICU accuracy study is currently underway with a further enhanced version of GlucoClear CGM.

**GluMetrics:** The GluCath® Intravascular CGM System from GluMetrics, Inc. measures blood sugar by the quenched chemical fluorescence technique. The sensor is deployed through a radial artery catheter and optical measurements are made every 10 seconds. A five minute rolling average of analyzed glucose is the displayed result. The device is intended for use in post-cardiothoracic surgery patients in whom glycemic control is indicated. The device has been tested on 70 subjects in the critical care setting, comparing results from the device against hourly arterial samples tested on the blood gas analyzers of 4 surgical ICUs (results forthcoming).

**GlySure:** GlySure’s intravascular blood glucose sensor uses optical fluorescence technology. Data are collected every few seconds and generate a continuous trend of blood glucose from the sensor, which is placed in the external jugular vein. The device is intended for use in management of glycemic control of intensive care patients. Results from initial clinical evaluation were published at the ISICEM meeting in Brussels 2012, and a poster updating that progress was accepted for publication at the World Intensive Care Congress in Durban in August/September 2013. The device is not yet available commercially.

**Maquet:** EIRUS® from Maquet Critical Care is a device for continuous monitoring of blood glucose and blood lactate in hospitalized patients, including ICU patients and patients during surgery. The system requires a special, multipurpose central venous catheter and the measuring principle is microdialysis. The glucose and lactate concentrations in the dialysate are in equilibrium with central venous blood concentrations and are analyzed every second. The measures are displayed every minute on the screen, with a delay of 5 minutes from actual sampling to displayed result. Accuracy of the device has been tested clinically in a critical care setting. As reference, arterial blood gas samples were analyzed in a blood gas analyzer. Paired glucose samples (607 and 994 paired samples) were analyzed using Clarke Error Grid and Bland-Altman analysis^,^. Lactate measurements (1,601 paired samples) were analyzed using correlation coefficient and Bland-Altman analysis.

- **Medtronic:** The Sentrino® System was designed to address the unique needs of critical care patients with a highly innovative design and unique ability to integrate into clinical protocols. The minimally invasive, subcutaneous sensor was customized for the critical care patient and inserts quickly and easily with low complication rates. Redundant sensing technology optimizes signal reliability for more accurate visibility of glycemic variability. A novel drug interference rejection technology ensures minimal interference with the wide array of pharmaceuticals used in critical care. The Sentrino CGM System is easily configured by clinicians and easily integrates into existing clinical workflow. The compact, flexible system is easy to transport with the patient.

**Menarini:** The GlucoMen®Day device (A Menarini Diagnostics) measures blood sugar by the microdialysis technique, combining an intravascular probe with an external GOx-based amperometric biosensor, highly sensitive and immune to a wide range of endogenous/exogenous interfering chemicals. Dialysate samples downstream of the probe are analyzed each minute, with 2 minutes delay from sample to displayed result, wirelessly transmitted to a bedside monitor. The suggested indication of our device is critically ill patients. The device is presently investigational, and has been tested for 72 hours in T1DM subjects against venous standard, using point and trend accuracy metrics (for example, Bias Plot, CEG, CG-EGA) to report performance.

**Optiscan:** The OptiScanner^®^ measures blood sugar in plasma using mid-infrared spectroscopy. Its fluidics systems withdraw a sample of non-diluted whole blood every 15 minutes, applies heparin (without any heparin exposure to the patient), and centrifuges the sample to plasma. The OptiScanner^®^ requires no calibration over several years. Clinical research supports usage of the OptiScanner^®^ on in-hospital diabetics, acute myocardial infarction patients, and medical/surgical ICU patients. Blood access can be obtained through standard venous catheters: a central venous catheter, a peripheral intravenous access, or a peripherally inserted central catheter. OptiScanner^®^’s glucose accuracy has been published in three peer-reviewed articles.
